# CNS-Spleen Axis – a Close Interplay in Mediating Inflammatory Responses in Burn Patients and a Key to Novel Burn Therapeutics

**DOI:** 10.3389/fimmu.2021.720221

**Published:** 2021-09-01

**Authors:** Noorisah Khan, Supreet Kaur, Carly M. Knuth, Marc G. Jeschke

**Affiliations:** ^1^Ross Tilley Burn Centre, Sunnybrook Health Sciences Centre, Toronto, ON, Canada; ^2^Institute of Medical Science, University of Toronto, Toronto, ON, Canada

**Keywords:** trauma, CNS, spleen, burns, sepsis

## Abstract

Severe burn-induced inflammation and subsequent hypermetabolic response can lead to profound infection and sepsis, resulting in multiple organ failure and high mortality risk in patients. This represents an extremely challenging issue for clinicians as sepsis is the leading cause of mortality in burn patients. Since hyperinflammation and immune dysfunction are a result of an immune imbalance, restoring these conditions seem to have promising benefits for burn patients. A key network that modulates the immune balance is the central nervous system (CNS)-spleen axis, which coordinates multiple signaling pathways, including sympathetic and parasympathetic pathways. Modulating inflammation is a key strategy that researchers use to understand neuroimmunomodulation in other hyperinflammatory disease models and modulating the CNS-spleen axis has led to improved clinical outcomes in patients. As the immune balance is paramount for recovery in burn-induced sepsis and patients with hyperinflammatory conditions, it appears that severe burn injuries substantially alter this CNS-spleen axis. Therefore, it is essential to address and discuss the potential therapeutic techniques that target the CNS-spleen axis that aim to restore homeostasis in burn patients. To understand this in detail, we have conducted a systematic review to explore the role of the CNS-spleen axis and its impact on immunomodulation concerning the burn-induced hypermetabolic response and associated sepsis complications. Furthermore, this thorough review explores the role of the spleen, CNS-spleen axis in the ebb and flow phases following a severe burn, how this axis induces metabolic factors and immune dysfunction, and therapeutic techniques and chemical interventions that restore the immune balance *via* neuroimmunomodulation.

## Introduction

Severe burns (>20% total body surface area) are one of the most devastating traumas and are a global public health concern (1–3). The World Health Organization estimates that burn injuries account for approximately 11 million injuries and 180,000 deaths per annum globally (4). Severe burns trigger a complex pattern of responses, such as inflammation and stress hormone signaling induced hypermetabolism, that persists for years after the initial injury. One of the initial consequences is a systemic inflammatory response, which occurs within hours after the initial insult to promote recovery. However, the inflammatory response to burn injury, unlike other forms of trauma, is highly dysregulated and may be repeatedly activated throughout recovery. Repeated and uncontrolled immune activation and stress hormone signaling greatly increases the susceptibility to infections and sepsis that often lead to multiorgan failure and have a high associated risk of mortality in patients. Hypermetabolism, sepsis, and consequent multiorgan failure are mediated through the immune system. Thus, immune dysfunction is a major contributor to these adverse outcomes. This poses a significant challenge to patient care, as controlling sepsis in burn patients requires a multifaceted approach that differs from sepsis in the general population. Although sepsis is the primary cause of death in burn patients that survive the initial phase of burn shock, there are currently no effective interventions as the sepsis syndrome remains poorly understood.

The CNS-spleen axis has a central role in immune function modulation with its sympathetic and parasympathetic pathways. In light of this, we propose that the CNS-spleen axis plays an important role in mediating the inflammatory response in burn patients. Given the challenges noted above, there is a growing need to understand the key players of the inflammatory response in burn patients to determine effective treatment strategies. Furthermore, we suggest that targeting this underappreciated CNS-spleen axis may lead to identifying new treatment strategies, which may improve the outcome of septic burn patients. The spleen plays a prominent role in blood filtration and regulating systemic immune responses (5). The spleen is unique to other secondary lymphoid organs given that it filters debris directly from the blood rather than through lymphatic drainage. Circulating blood first enters the splenic artery and is dispersed into the marginal zone (MZ), which separates the white pulp (WP) from the red pulp (RP). The MZ acts as a connection between circulating blood and immune cells. Macrophages within the MZ express a diverse set of surface markers to capture and process antigens to present to B cells (6, 7). Surrounding the central arterioles of the spleen is the WP, which is composed of the periarteriolar lymphoid sheath (PALS) and follicles. T cells and B cells are concentrated in the PALS and follicular zones, respectively, where they respond to presented antigens and coordinate the innate and adaptive immune responses. On the opposite side of the MZ is the RP, which is the primary site of blood filtration. Here, RP macrophages play a crucial role in filtering aged, dead, or opsonized cells from systemic circulation (6). Furthermore, myeloid cells, including granulocytes, monocytes, and macrophages are primarily located within the RP region (8). Granulocytes like neutrophils, eosinophils, basophils, and mast cells are mainly localized in the RP region, with some cells in transition through the MZ into RP (9). When neutrophils are recruited to inflammatory sites, they have short life spans and originate from the bone marrow (10). A neutrophil-intrinsic program, like a timer, allows neutrophils to mount an efficient defense while preserving vascular health (11). However, neutrophil activity doesn’t rise homogeneously throughout the entire hematopoietic system (10). Instead, the marrow-derived neutrophils that are located closest to the inflammation site migrate, rather than marrow-derived neutrophils that are further away in the body (10). Monocytes develop in the bone marrow from a common myeloid/dendritic cell progenitor and continuously migrate into blood and spleen as mature cells (12). When monocytes enter distant organs, they differentiate into macrophages upon inflammatory insult. Swirski et al. identified that there are also bona fide undifferentiated monocytes (Ly-6C^high^) that reside in the spleen and that are larger in number than their blood monocyte equivalents (13). These reservoir monocytes assemble in clusters in the cords of the subcapsular RP and are different from macrophages and dendritic cells (13). Splenic Ly-6C^high^ monocytes can migrate from the subcapsular RP to distant sites under inflammation conditions such as ischemic stroke, even though there is no change in the bone marrow monocytes (13). This shows that the splenic monocytes increase motility, exit the spleen in bulk, and participate in rapid wound healing (13). Splenic monocytes are deployed through a variety of receptors on monocyte surfaces such as angiotensin type I (AT-1) and angiotensin type II (AT-2) receptors (13).

Not only is the spleen highly vascularized, but it is also extensively innervated. This allows for a crucial communication pathway between the CNS and the spleen to mediate systemic inflammatory responses. Specifically, the CNS aids the spleen in modulating inflammatory responses through both branches of the autonomic nervous system, *via* the sympathetic nervous system (SNS) and parasympathetic nervous system (PNS) pathways (14, 15). The spleen is innervated by efferent sympathetic and splenic fibers that originate in the celiac-superior mesenteric plexus ganglion. Most nerve fibers that innervate the spleen are catecholaminergic, hence under the control of the SNS. Indeed, tyrosine hydroxylase (TH)-positive sympathetic fibers are often observed within proximity to lymphocytes in the WP and macrophages in the RP, as demonstrated by immunostaining (14, 15). Furthermore, it is presumed that direct synaptic contact exists between sympathetic axons and B cells in the WP (15, 16). Activation of the SNS suppresses the activity of innate immune cells while promoting the activity of adaptive immune cells (17). Through the sympathetic fibers, the primary sympathetic neurotransmitters epinephrine and norepinephrine primarily bind to the beta-2 adrenergic receptors (β_2_AR) on immune, vasculature, and connective tissue cells. This activates splenic cytokines such as Tumor necrosis factor α (TNFα), and Human mobility group B1 (HMGB1), which increases pro-inflammation in the body. In fact, not only does the SNS communicate with the spleen, but the cytokines released by the spleen also communicate with the SNS in a negative feedback loop (18, 19). Neurotransmitters released by the neuronal network affect the immune cell activity by either inhibiting or inducing inflammation (18, 20, 21). The classic opposing branch of the autonomic nervous system that regulates communication from the CNS to the spleen is through the PNS, specifically through its primary neurotransmitter acetylcholine (ACh) and the vagus nerve. This is known as the cholinergic anti-inflammatory pathway (22). Neuronal tracing studies have demonstrated the absence of direct cholinergic innervation by vagus nerve fibers in the spleen (17). However, the cholinergic pathway mediates cytokine production through a two-step neuronal process in which information is transmitted from the preganglionic neurons originating in the vagus nerve and projected through the postganglionic neurons in the splenic nerve (17). Moreover, ACh derived from the vagus nerve inhibits the release of proinflammatory cytokines in the spleen and thereby decreases systemic inflammation (17). The actions of ACh are heavily dependent on the nicotinic acetylcholine α7 subunit receptor (23). Therefore, not only do sympathetic nerve fibers control spleen immune cell function but these effects can also be attributed to the cholinergic anti-inflammatory pathway *via* the vagus nerve.

As a result, many cross-communicative networks exist between the SNS and PNS pathways to maintain an immunologic balance in the body. One network is cytokine receptor regulation of immunity, specifically, the cytokines released through SNS neurotransmitter, norepinephrine, can influence the function of neurons in the PNS pathway *via* cognate receptors on the neuronal cell surfaces (6). These cytokines such as interleukin-1β (IL-1β), interact with their associated receptors as well as the N-methyl-d-aspartate (NMDA-R) receptor (24). These receptors in turn activate the PNS pathway and secrete ACh to inhibit these cytokines and subsequent hyperinflammation by way of the vagus nerve. Furthermore, T cells are also able to modulate the SNS pathway by reducing sympathetic fibers in the spleen and affecting the pituitary-adrenal axis in the CNS through diminished hypothalamic norepinephrine concentrations, thereby decreasing cytokine release (21).

A second network is the catecholaminergic regulation of immunity. Sensory neuron activation by the soleus muscle causes activation of catecholaminergic signaling to dorsal blood vessels of the fifth lumbar cord (25). This is mechanistically related to increased expression of cytokines such as CCL20 which leads to increased accumulation of pathogenic T cells and inflammation (25). After inflammation, the release of norepinephrine from catecholaminergic nerve endings in the SNS modulates the activity of invariant natural killer T cells (iNKT) (25). This modulation involves suppression of T_H_1-type release of cytokines such as interferon-γ and enhancement of T_H_2-type cytokine release, including IL-10, leading to immunosuppression, as done by the PNS (25).

A third network is Pavlovian conditioning and reward system regulation of immunity which functions through catecholaminergic signaling. Specifically, Goebel et al. found that after repeated pairing of a flavored drink (conditioned stimulus) and cyclosporin A (unconditioned stimulus), the drink alone mimicked the effects of cyclosporin A and resulted in suppression of immune function, by way of the PNS, including impaired T_H_1 cytokine production and reduced T cell proliferation (26). Furthermore, by stimulating dopamine receptors in a brain reward region (ventral tegmental area), there was an enhanced peripheral immune response as indicated by increased phagocytic activity of dendritic cells and macrophages, and activation of monocytes (27).

A fourth cross-communicative network is through the axon reflex-like regulation of immunity. An axon-reflex is a type of reflex in which receptor activation of peripheral axonal branches of sensory neurons triggers signals transmitted to the bodies of the neurons and diverted to other peripheral axonal endings with the generation of a response (28). Specifically, in an axon reflex-like way, sensory neurons specialized in pain perception (nociceptors) modulate the local immune response to inflammation caused by specific pathogens such as *Staphylococcus aureus*. The pathogen activates nociceptors by releasing N-formyl peptides and pore forming toxin α-hemolysin, inducing action potentials (25). In turn, these neurons release neuroimmunomodulatory peptides, including calcitonin gene-related peptide, galanin, and somatostatin at the site of infection, which inhibit innate immune activation *via* interaction with neutrophils, monocytes, and macrophages like the PNS (29).

**Figure 1:**
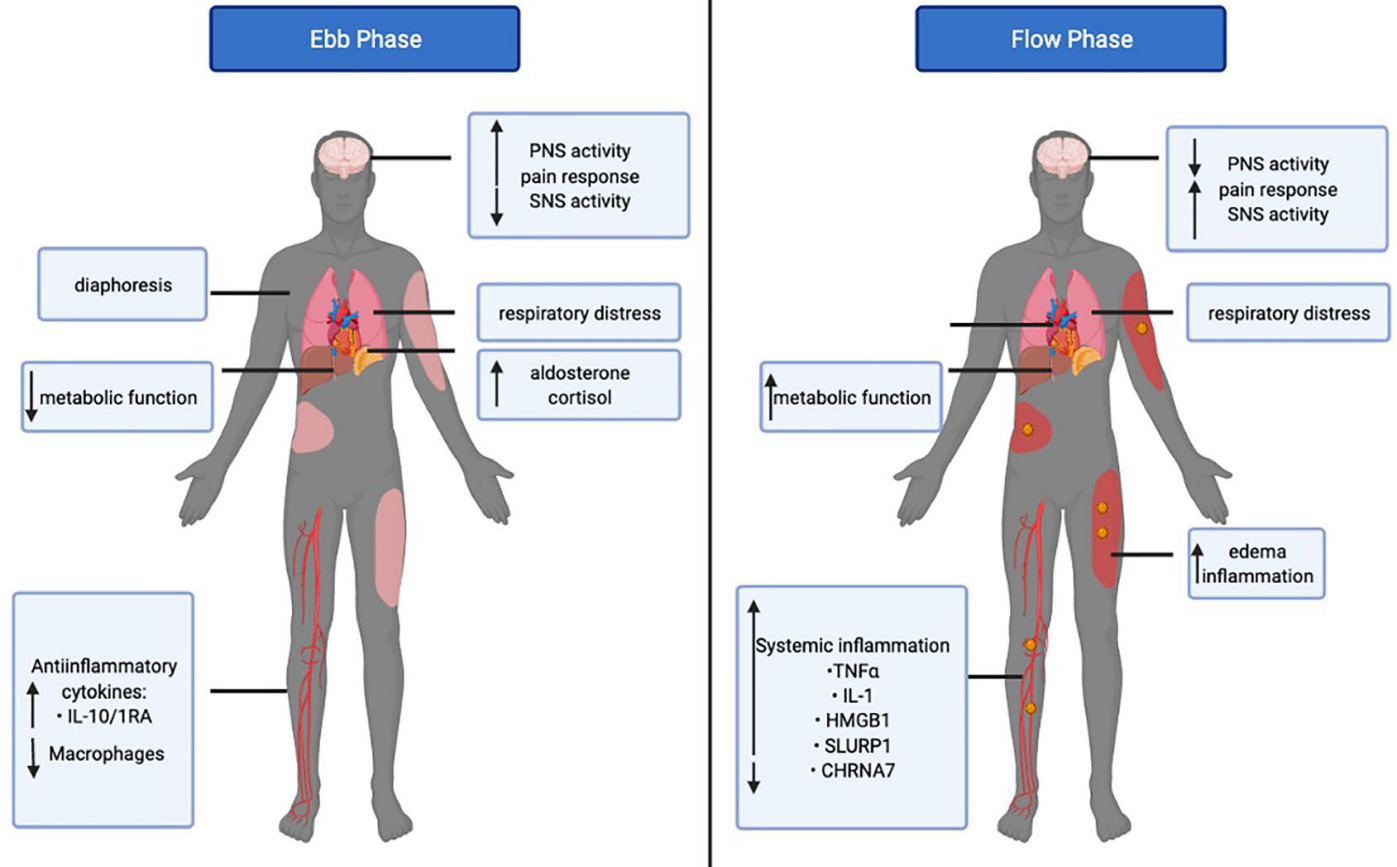
The bi-directional communication between the central nervous system (CNS) and the spleen. The parasympathetic nervous system (PNS) uses acetylcholine and the vagus nerve to decrease burn-induced inflammation *via* the spleen. The sympathetic nervous system (SNS) uses norepinephrine and the hypothalamus, pituitary, and adrenal axis (HPA axis) to increase burn-induced inflammation *via* the spleen. In turn, the CNS is regulated by the cytokines released from the immune cells of the spleen in a negative feedback loop.

Therefore, it seems evident that the CNS-spleen axis plays a crucial role in regulating systemic immune responses (**Figure 1**). It has been postulated that patients with inflammatory diseases have a dysfunctional autonomic nervous system, leading to the overproduction of proinflammatory cytokines, such as TNFα and IL-1β. Targeting this axis has proven to be beneficial in patients with ongoing inflammatory conditions, such as Crohn’s disease, rheumatoid arthritis, and sepsis (19, 30, 31). Given the clinical efficacy in treating patients with chronic inflammatory conditions by modulating the CNS-spleen axis, it is intriguing to consider whether restoring autonomic-immune homeostasis may be effective in treating the septic burn population.

This review is the first to highlight the importance of the CNS-spleen axis in modulating the response to sepsis in the severe burn patient population. We will first discuss the role of the CNS-spleen axis during the ebb and flow stages of recovery. Next, we distinguish the changes that are evident in septic burn patients that survive compared to those that succumb to their injury with regards to the CNS-spleen axis. Finally, we will address and discuss the potential therapeutic techniques that target the CNS-spleen axis, which has demonstrated efficacy in inflammatory diseases but has not yet been explored in the context of sepsis and burns. Overall, this review aims to 1) critically analyze the potential contribution of the CNS-spleen axis in modulating the immune response in the severe burn patient population, and 2) discuss the therapeutic advances made thus far along with areas of improvement in targeting the CNS-spleen axis to treat the septic burn population.

## The Role of the CNS-Spleen Axis During the Post-Burn Hypometabolic “Ebb” Phase

**Figure 2:**
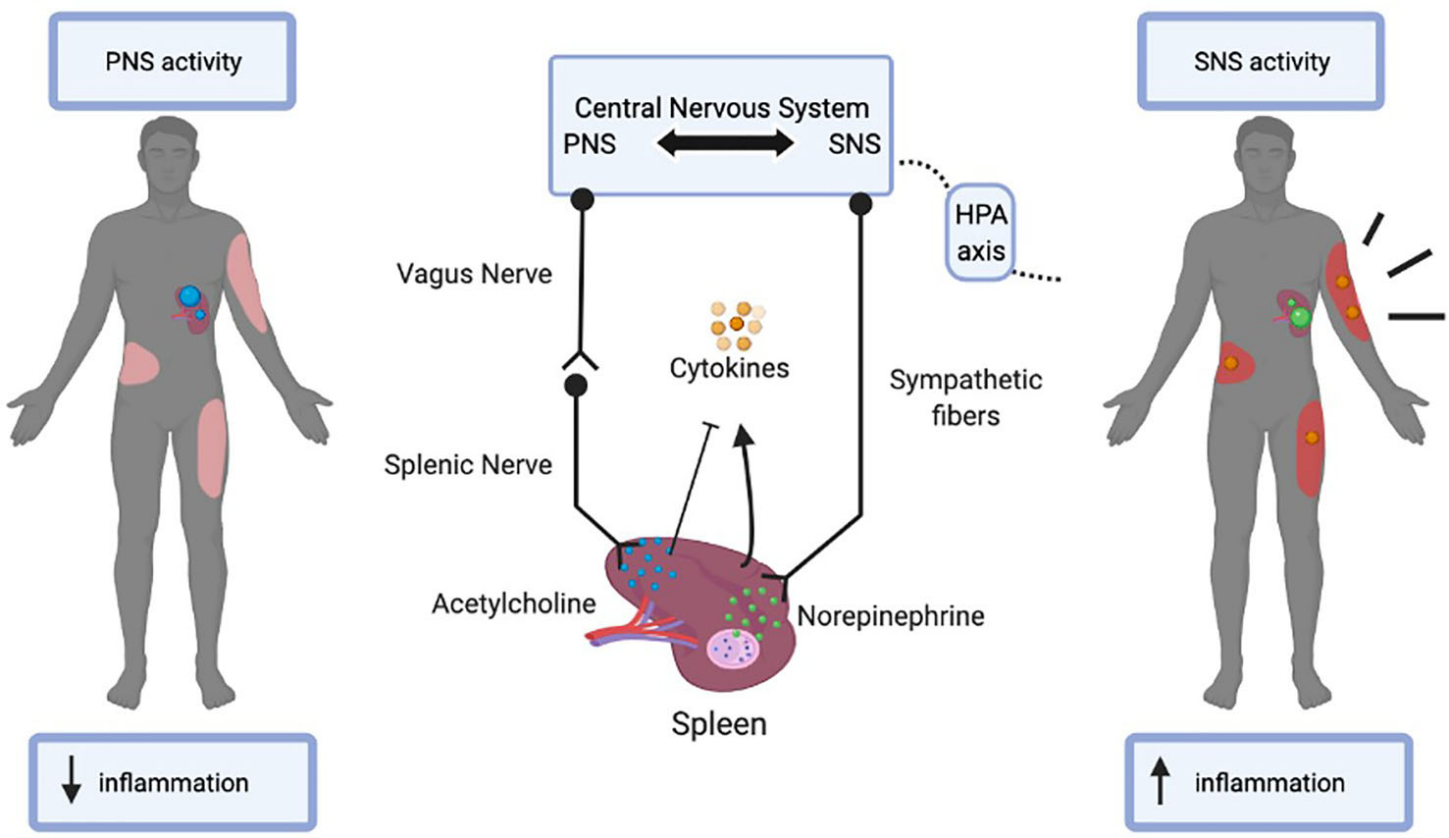
The Ebb and Flow phases of burn injury. The Ebb phase lasts for 24-48 post-burn injury whereas the flow phase may last up to 2 years. The figure illustrates the differential systemic and metabolic response in the ebb versus flow phase in burn patients. Tumor necrosis factor-alpha (TNFα), Interleukin 1 (IL-1), High mobility group box 1 (HMGB1), Secreted Ly-6/uPAR-related protein 1 (SLURP1), Cholinergic Receptor Nicotinic Alpha 7 Subunit (CHRNA7).

After a severe burn injury, patients undergo a biphasic metabolic response to burn injury. The acute response is known as to as the ebb (or shock) phase and the chronic response is referred to as the flow phase (**Figure 2**) (32, 33). The ebb phase develops immediately post-burn, lasting approximately 72-96h (34). The ebb phase is characterized as a hypodynamic state whereby respiratory distress occurs and the metabolic rate is depressed to reduce energy depletion (32). The ebb phase is present for a limited duration and is a period that dictates whether a patient will survive or succumb to burn injury-associated complications. Chen et al. previously showed that patients demonstrating abnormally high levels of proinflammatory IL-1β and decreased macrophages at the site of injury are highly susceptible to the development of sepsis (35). Furthermore, septic patients also had increased anti-inflammatory plasma cytokines such as interleukin-10 (IL-10) and interleukin-1RA (IL-1RA) (35). The Septic Predictor Index (SPI), which describes the ratio of IL-1β from stromal vascular fraction-derived macrophages and macrophage proportion (i.e., the proportion of CD14+ IL-1β+ cells/proportion of CD14 high CD16 low cells), was generated for the determination of macrophage and IL-1β production at the site of injury (35). All patients who developed sepsis had SPI values of >0.5, meaning that their sepsis had a later onset (<12 days). The onset of sepsis in patients SPI values of >1 occurred within 12 days post-injury, whereas a delayed onset of sepsis (>12 days)_occurred in patients with SPI values between 0.5–1 (35). During the ebb phase, some inflammatory mediators, such as cortisol, catecholamines, and aldosterone are elevated immediately post-burn (36). Furthermore, serum cytokines such as IL-6, IL-8, granulocyte colony-stimulating factor (G-CSF), and monocyte chemoattractant protein-1(MCP-1) demonstrated up to a 2000-fold increase immediately upon burn trauma (36). Mast cells (MC) are key contributors in blood coagulation as well as innate and acquired immunity (37). Evidence suggests that MCs play an important role in tissue repair and while MCs initially promote healing, they can be detrimental if activated chronically (37). Burn trauma has a direct effect on MCs leading to the secretion of histamine (38). Bankova et al. provided evidence that MC degranulation is an instantaneous response following thermal injury in the ebb phase (39). This leads to an increased xanthine oxidase activity and enhanced production of reactive oxygen species (ROS); the latter being produced after burns through differing mechanisms (39). Nonetheless, sepsis and extreme hyperinflammation do not occur in the ebb phase of burn injury (32). Concerning the CNS-spleen axis, there is decreased SNS activity and increased PNS activity in efforts to decrease inflammation. Under healthy conditions, the SNS and PNS work in opposition to the body to maintain homeostasis. However, during this bi-directional communication between the CNS and the spleen, the SNS and PNS work complementary to one another (40). During the ebb phase of burn injury, sepsis is not evident, however when the ebb phase is overcome by the flow phase, sepsis becomes a grave challenge (3).

## The Role of the CNS-Spleen Axis During the Post-Burn Hypermetabolic “Flow” Phase

The post-burn hypermetabolic “flow” phase occurs after the ebb phase, which leads to an increased metabolic turnover and activation of the immune system and may last up to several years (**Figure 2**). Concerning the CNS-spleen axis, SNS activity is persistently elevated in the flow phase (3). As a result, increased circulating concentrations of cytokines and persistent inflammation may contribute to the development of sepsis (32). Given the increase in SNS activity, this results in a hypermetabolic stress response, which causes severe catabolism, immune dysfunction and profound physiological perturbations that may also contribute to the development of sepsis (3). It is evident that these changes in the body in response to the flow phase of burn injuries are a major cause for concern in patient care and if left untreated, physiologic exhaustion may occur and the injury becomes fatal (3). Sepsis in the general population has many differences from sepsis in the burn population as the primary barrier (skin) is absent, and infection persists as long as the skin is absent (41). Additionally, the systemic dysfunctional state decreases intestinal barrier permeability, increasing the risk of sepsis (42). In severe burn patients, the SNS stimulates pro-inflammatory responses leading to persistently high circulating cytokine concentrations, which cause hyperactive inflammation for which the spleen plays a major role in regulating. The CNS-spleen axis is therefore a major target of clinical research in trying to decipher the ability to control the body’s response to immunological issues such as burn-induced inflammation and sepsis. A severe burn injury results in enhanced systemic inflammation, and to effectively manage this, it is important to decipher the role of the CNS in modulating the systemic levels of inflammation and its role in maintaining homeostasis. In the flow phase of burn injuries, the spleen is one of the major sources of mediators that contribute to an exaggerated inflammatory response. Myeloid-derived suppressor cells (MDSCs) are immature myeloid cells with simultaneous proinflammatory and immunosuppressive properties (43). MDSCs are potent producers of TNFα and ROS, which are released from splenic macrophages in response to injury (43). TNFα is a necessary and sufficient mediator of local and systemic inflammation with regards to burns (44–47). TNFα enhances and prolongs an inflammatory response by activating other cells to release pro-inflammatory cytokines such as IL-1 and HMGB1 (48). IL-1 is released by macrophages, lymphocytes, and monocytes during infection, injury and inflammation (49). HMGB1 is secreted into the extracellular space and acts as a proinflammatory cytokine, like IL-1. As such, a persistent elevation in HMGB1 and IL-1 cytokine concentrations are evident in the flow phase of burn patients (49, 50). TNFα also activates other cells to release mediators such as eicosanoids, nitric oxide, and ROS, which further promote inflammation and tissue injury (51). In the flow phase, persistent MDSC expansion exerts harmful effects by propagating persistent inflammation while dampening the adaptive immune response *via* T-cell suppression (43).

The α7 nicotinic acetylcholine receptor (CHRNA7) is important in the signaling performed by the vagus nerve to inhibit proinflammatory cytokines. ACh, released by the vagus nerve and immune cells, acts as a ligand for the CHRNA7 receptor and induces inhibition of proinflammatory cytokines in the spleen. Holmes et al. found a >70% reduction of protein levels of the CHRNA7 in burn patients despite significantly elevated ACh (52). Additionally, proinflammatory cytokines were persistently active in burn patients as ACh signaling by the vagus nerve was not effective. Furthermore, the gene and protein levels of an endogenous allosteric modulator of CHRNA7, secreted mammalian Ly-6/urokinase-type plasminogen activator receptor-related protein-1 (SLURP1), were significantly elevated in efforts to elevate CHRNA7 numbers although to no avail (52). In the flow phase, although ACh levels were significantly elevated, CHRNA7 protein levels remained low, and inflammation was not impeded. Furthermore, SLURP1 failed to increase CHRNA7 levels, thereby leading to excessive proinflammation.

Additionally, extramedullary hematopoiesis occurs in the flow phase in which hematopoietic stresses mobilize hematopoietic stem cells from the bone marrow to the spleen (53). Splenic tissue weight increased significantly at post-burn day 7 in mice, indicating splenomegaly, presumably to support extramedullary hematopoiesis (54). Furthermore, MDSC expansion is associated with extramedullary hematopoiesis, suggesting that erythropoiesis in the spleen and liver are components of emergency myelopoiesis (43).

It is important to note that much of the criteria for sepsis in the general population is already present in the burn patient population, however, without septic infection (41). Therefore, treatment for sepsis within the burn patient population is difficult given the lack of specific and effective diagnostic criteria. Moreover, classic antibiotic cocktails used for sepsis in the general population are ineffective in burn patients. Therefore, prior to discussing therapeutic options between the general septic and the septic burn populations, it is important to distinguish the physiological changes that are evident in septic burn patients that survive compared to those that succumb to their injury with regards to the CNS-spleen axis.

## Survival *vs.* Death in Septic Burn Patients

The post-recovery phases of burn injuries in patients who survive septic burns are not fully independent of post-septic symptoms. Post-sepsis syndrome is a condition that affects sepsis survivors, in which they have a range of physical, psychological, and emotional challenges while recovering. The categories of long-term effects of sepsis include immunometabolic, neurocognitive, psychiatric, and physical derangements (55). These effects lead to enhanced mortality risk and severely impaired quality of life. In burn patients, the SNS is hyper-activated in post-sepsis syndrome, which leads to increased levels of proinflammatory cytokines such as HMGB1 (55). Patients and murine models who have survived burn-induced sepsis and inflammation have systemic changes such as extreme splenocyte dysfunction (56). HMGB1 plays a key role in mediating the immune dysfunction of splenocytes in sepsis survivors (56). Because of excess cytokines such as HMGB1, sepsis survivors have altered adaptive immune cells, such as T cells and B cells, that do not function normally in eradicating further systemic inflammation (56). Specifically, the population of cytotoxic T cells (CD8+ T cells), helper T cells (CD4+ Th cells), regulatory T cells (Treg cells), and B cells declines in a post-septic mouse model of sepsis as compared to sham controls (57).

Since much of the criteria for sepsis in the general population is already present in the burn patient population, it is important to explore the pathophysiological changes evident in the general post-septic populations (41). In a murine model of sepsis-induced by experimental cecal ligation and puncture (CLP), mice developed splenomegaly (58). This was due to a higher-than-normal activity of the spleen in secreting inflammatory cytokines such as TNFα and HMGB1. This occurred due to the sympathetic fibers innervating the spleen in the CNS-spleen axis and the SNS signaling to the spleen *via* norepinephrine to secrete more cytokines. Serum HMGB1 levels in septic mice were found to be significantly elevated for 4‐6 weeks, and administration of an anti‐HMGB1 monoclonal antibody significantly attenuated splenomegaly as well as splenocyte priming since the effects of cytokines produced *via* the SNS were inhibited (58). It is possible that in survivors of sepsis, HMGB1 may be a mediator of the initial over-reactivity of the immune response, but later leading to late immunosuppression in sepsis survivors. Therefore, HMGB1 is hyperactivated in post-septic survivors due to the overactive SNS and lack of effective control by the PNS. These data could be implicated towards the septic burn population however further research is needed to identify whether targeting HMGB1 would effectively mitigate splenomegaly in this population.

Jeremias et al. have identified the key role of ACh in the inflammatory response 15 days following CLP-induced sepsis in a murine model. The authors developed a murine model of dysfunctional ACh transporters (ACh KO) to determine the post-septic effects on the PNS response of the CNS-spleen axis. ACh KO mice with CLP-induced sepsis demonstrated a decreased CD8+ cytotoxic T cells and CD4+ Th and an increase in Th17 cells compared to the control (57). Th17 cells are pro-inflammatory cells that secrete inflammatory cytokines such as interleukin-17A, IL-21, and IL-22 (59). Since the ACh transporter was not present, vagus nerve activity was not being carried out properly by the PNS and as such, proinflammatory cytokine levels were high due to lack of inhibition of the SNS. This led to increased inflammation in post septic mouse survivors compared to the sham controls because of the downregulated PNS.

Burn survivors and post-septic deceased patients have multiple similarities in the bodily changes that have occurred because of septic burns. However, post-septic deceased patients have some distinguishing characteristics. Patients who die as a result of burn-induced systemic sepsis have a marked loss of noradrenergic nerves, which occurs due to the apoptosis of splenic cells (50). Nerve loss in patients may be attributed to reduced availability of neurotrophic factors that support sympathetic neurites or enhanced production of chemicals and inflammatory cytokines, which injure nerves (50). For example, TH, which is the rate-limiting enzyme in the synthesis of norepinephrine, is also lacking in patients who die from general sepsis (50). Since norepinephrine synthesis is decreased, the SNS pathway communication is hindered, and inflammation is low in deceased septic patients. WP is also greatly reduced in patients who die as a result of septic burns (60). Moreover, germinal centers, the area responsible for initially mounting an immune response, are also greatly reduced or completely lacking in post-septic burn patients (61). These factors indicate a strong correlation of CNS-spleen axis response in modulating the inflammatory response in sepsis alone or burn sepsis patients that improves survival. Although a dearth of literature is present for general post-septic survivors and deceased models/patients, further research is required to disseminate the alterations relative to burn-induced sepsis. Moreover, it is imperative to address the potential therapeutic techniques that target the CNS-spleen axis, which has demonstrated efficacy in inflammatory diseases, such as general sepsis, but have not yet been explored in the context of burn-induced sepsis.

## Therapeutic Techniques for Neuroimmunomodulation in Burn-Induced Sepsis

The CNS-spleen axis may be of great importance in combating burn-induced sepsis as it regulates systemic inflammation. Various novel therapies that target the CNS-spleen axis exist for the treatment of the general sepsis population. These therapies may be effective in the septic burn population too. Here, we will discuss the potential use of vagus nerve stimulation (VNS), agonists and antagonists of major molecules in the CNS-spleen axis, approaches targeting proinflammatory cytokines and approaches targeting catecholamines to mitigate burn-induced sepsis (**Table 1**).

**Table 1:** Techniques for the treatment of Burn induced Sepsis and inflammation.

Categories of Major Treatment Strategy	Type of Treatment Strategy	Description	Study Conducted in
Vagus Nerve Stimulation	Vagus Nerve stimulation	Similar to carotid sinus massage in which a pressure point on the neck is pressed for 5-7 seconds	Mice (62)
	Transcutaneous vagus nerve stimulation	Electrically stimulates the vagus nerve	Mice (63)
	CNI-1493	Vagus nerve stimulator	Mice (64)
	Amiodarone and MSH	Vagus nerve stimulator	Mice (65)
Agonists and antagonists of major mediators in the CNS-spleen axis	CNI-1493	Inhibitor of macrophage activation and TNF release	Mice (64)
	Amiodarone and MSH	Inhibitor of TNF synthesis, and suppresses edema	Mice (65)
	GTS-21	A small molecule agonist for CHRNA7 and reduces sepsis	Mice (66)
	CHRNA7 agonists	Reduces sepsis	Mice (67)
	α-chemokine receptor inhibitors	Decreases inflammation by inhibiting cytokines	Mice (68, 69)
	Stearoyl lysophosphatidylcholine	Stimulates neutrophils to eliminate invading pathogens Suppresses HMGB1 release from macrophages	Mice (70)
Approaches targeting proinflammatory responses	Antibodies against proinflammatory cytokines	Anti-TNF decreased inflammation Anti-HMGB1(ethyl pyruvate) decreased inflammation	Mice (41, 52, 71, 72)
Catecholamines	Epinephrine, Norepinephrine, and dopamine	Along with cortisol decrease inflammation	Mice (73)

### Vagus Nerve Stimulation

VNS is the most extensively studied and successful neuroimmunomodulation technique in treating burn patients (71, 74–76). As discussed previously, the vagus nerve plays a key role in suppressing inflammation as it inhibits pro-inflammatory cytokine release through ACh. Vagus nerve stimulation leads to the release of Ach in organs of the reticuloendothelial system, including the liver, heart, spleen, and gastrointestinal tract. ACh interacts with α-bungarotoxin-sensitive nicotinic CHRNA7 receptors on tissue macrophages, which inhibit the release of TNFα, IL-1, HMGB1 and other proinflammatory cytokines (77). Researchers have identified several VNS techniques, which support the use of VNS in the clinical setting for sepsis patients (60). Experimental studies have shown that invasive VNS, involving a direct electrical stimulation, significantly reduces pro-inflammatory cytokines (62). Furthermore, systemic inflammation can be attenuated by using a non-invasive method of VNS that is based on carotid sinus massage used for the treatment of rhythmic disturbances (78). This method of VNS involves massaging the vagus nerve pressure point along the neck for 5-7 seconds (79). VNS has been shown in a dose-dependent manner to reduce systemic TNFα levels during lethal endotoxemia (78). Another method of VNS is transcutaneous VNS, which acts on the afferent auricular branch of the vagus nerve through electrodes placed on the skin of the left ear (80). The electrical signal, starting in the auricular branch of the vagus nerve, reaches the nucleus tractus solitarius, which is a crucial structure that projects to a variety of brain areas (81). One such area is the locus coeruleus, which is a primary source of norepinephrine (81). A study by Huston et al. determined the effects of transcutaneous VNS on serum HMGB1 levels and survival in murine sepsis (63). Following 3 weeks of transcutaneous VNS treatment, the survival rate improved by 30% in comparison to controls. VNS also attenuated serum HMGB1 levels by 50% as compared to the control mice (63). Transcutaneous VNS carried out through electrodes inhibits norepinephrine and subsequently reduces pro-inflammatory cytokine production through the CNS-spleen axis (63). The therapeutic potential of transcutaneous VNS has not yet been assessed in burn septic models. However, its potential efficacy in this population makes transcutaneous VNS an intriguing avenue to explore.

There are several limitations to consider with the use of VNS treatments in septic burn patients, including the absence of healthy skin. Additionally, the hypermetabolic response persists far past wound closure, indicating that further treatment is necessary following the wound healing process. However, since VNS stimulation can attenuate HMGB1 levels and therefore reduce inflammation in septic patients, it may succeed in the burn sepsis population.

### Approaches Using Agonists and Antagonists of Major Mediators in the CNS-Spleen Axis

Agonists and antagonists are molecules that can target major mediators in the CNS-spleen axis. The goal in utilizing these pharmacologics is to alter the CNS-spleen axis activity to decrease pro-inflammatory cytokine production

Some of these molecules, namely, CNI-1493, Amiodarone and MSH, target cytokine production by interacting with the vagus nerve. CNI-1493, a tetravalent guanylhydrazone, is an antagonist towards macrophage activation and TNFα release in local and systemic inflammation models (64). Similarly, Amiodarone and α-melanocyte-stimulating hormone (MSH) are antagonists of TNFα synthesis *in vitro* and suppress the development of swelling and edema by decreased exposure to TNFα (65). CNI-1493 and Amiodarone both function as pharmacological stimulators of the vagus nerve (60, 65). Therefore, these treatments may be effective in the burn sepsis population.

Some molecules, rather than directly targeting the vagus nerve, focus on relieving inflammation through the cholinergic pathway, specifically through CHRNA7 receptors. GTS-21 is a small molecule partial agonist for CHRNA7, which has been used to study cholinergic anti-inflammatory mechanisms in mice (66). Treatment with GTS-21 in a mouse model of burn injury completely abolished mortality at 7-days post-burn in comparison to their untreated burn-injured counterparts, which demonstrated a 25% survival rate (66). GTS-21, along with other small molecules such as CNI-1493, which initiate signals in proximal components of the pathway in the CNS-spleen axis, were found to be beneficial in mediating the effects of sepsis and inflammation. However, clinical efficacy remains to be determined.

The CHRNA7 gene was originally found to be effective in controlling inflammation by modulating CHRNA7 expression on cytokine-producing cells during infection and tissue damage (67). CHRNA7 protein levels were found to be low in burn patients during hyperinflammatory states (52). Moreover, Holmes et al. assessed the impact of activating CHRNA7 on keratinocytes and found it to be a successful alternative to block inflammatory cytokine induction and HMGB1 release (52). In general, it has been shown that α-chemokine receptor antagonists increase survival in sepsis patients in a clinically relevant time frame (68). On the other hand, CHRNA7 agonists attenuate systemic inflammation and improve survival in experimental sepsis in murine models. One limitation of these treatments is that they also act as sedatives (69). However, given that these treatments work through the CNS-spleen axis, specifically through the PNS to decrease inflammation, activators of CHRNA7 may be promising for the burn sepsis population.

Apart from acting on the vagus nerve or cholinergic pathway, some molecules work directly on the innate immune cells. In particular, neutrophils are the most plentiful in the blood and are the first line of defense of the immune system (82). Stearoyl lysophosphatidylcholine (LPC), a class of chemical compounds that are derived from phosphatidylcholine, have recently been shown to be protective against lethal sepsis by stimulating neutrophils to eliminate invading pathogens by reducing reactive oxygen species (70). Stearoyl LPCs significantly attenuates circulating HMGB1 in sepsis by suppressing HMGB1 release from macrophages/monocytes (70). As such, neutrophils may be an indirect way to combat burn-induced sepsis.

### Approaches Targeting Proinflammatory Cytokines

Previously, anti-TNFα antibodies have been shown to reduce septic inflammation, however, TNFα antibodies have not been found to be successful in the late stages of sepsis given that antibodies are not effective if therapy is started after serum clearance of TNFα (45, 72, 83). However, researchers have found that the usage of antibodies targeting HMGB1, which is produced in the late stages of sepsis, is much more promising (44, 84, 85). Moreover, HMGB1 levels can also be controlled through ethyl pyruvate, a simple aliphatic ester of pyruvic acid, which has been shown to protect against lethal septic shock even if treatment begins after a TNFα response has been elicited (72). By administering ethyl pyruvate (24h after the onset of sepsis) mouse models were rescued from lethality despite suffering from the physiological effects of sepsis, leading to severe morbidity yet reduced mortality (72). Overall, antibodies against HMGB1 and administration of ethyl pyruvate have shown promising results in the sepsis population and could potentially be translated to the septic burn population.

### Approaches Targeting Catecholamines and Adrenergic Receptors

Catecholamines (epinephrine and norepinephrine) and dopamine act as vital hormones that are produced, in part, by the adrenal medulla and secreted into the circulation in response to stress or injury. Catecholamines influence and modulate sympathetic neural modulation of immune responsiveness (86). Specifically, epinephrine and other stress hormones such as cortisol have been found to inhibit cytokine synthesis and therefore, inhibit a pro-inflammatory response through the SNS pathway (86). Myeloid cells express α- and β-ARs while lymphocytes primarily express β-ARs (87). Catecholamine receptor signaling in macrophages has significant effects on the inflammatory response. Specifically, β-AR inhibition using β–blockers led to increased TNFα secretion following lipopolysaccharide (LPS) induced inflammation in murine models (88). Compared to the pro-inflammatory effect of the β-AR blockade, α-AR inhibition of murine macrophages using α-blockers led to decreased TNFα and IL-1β expression compared with LPS alone (87, 89). Together, these observations suggest that the differential roles of catecholamines on macrophages may depend on the targeted adrenergic receptor. Therefore, in septic burns, catecholamines and their associated receptors may be used to inhibit cytokine release and inhibit inflammatory responses.

## Conclusion

Sepsis is a major concern, not only in the general patient population but aggressively so in burn patients. The CNS-spleen axis pathway may be a crucial aspect to consider when developing treatments for burn patients as this bidirectional pathway communication leads to hyperinflammation in the body in response to burns and sepsis. Specifically, the SNS has been shown to increase pro-inflammatory cytokines in patients using epinephrine/norepinephrine, and the PNS has been shown to decrease inflammation in the body using ACh and the vagus nerve. Since targeting this axis has demonstrated clinical efficacy in patients with many ongoing inflammatory conditions (19, 30, 31), it is very intriguing to consider whether restoring autonomic-immune homeostasis may be effective in treating the septic burn population. Because sepsis onset occurs during the flow phase, it is a major target in burn patients. Methods such as VNS have shown promising results as they attenuate pro-inflammatory cytokines leading to decreased inflammation and increased survivability. However, further research is necessary to determine the effectiveness of using combination therapies in septic burn patients. Furthermore, it is also possible to activate neural anti-inflammatory mechanisms using small molecules that initiate signals in proximal components of the pathway in the CNS, such as CNI-1493. Important findings, such as with the use of GTS-21, has only been studied in murine burn sepsis models and should be translated to human patients to determine clinical efficacy (73). Furthermore, an important, but difficult implication in attempting to reduce systemic inflammation is deciphering whether beneficial inflammation is also being suppressed. Controlled inflammation by the CNS-spleen axis is a normal response to recruit immune cells to the site of infection or trauma to restore homeostasis and promote healing. Therefore, in burn injuries, inflammation should not be suppressed to the point in which wound healing is impaired. Although further research is required to find the balance between suppressing the beneficial versus harmful inflammation, research over the past few decades has made great advancements in developing methods to decrease burn-induced inflammation. Overall, the purpose of this review was to highlight the importance of the bidirectional communication of the CNS-spleen axis given the regulatory role in immunogenic responses, which can be targeted to reduce sepsis and inflammation. Failure of the body in regulating these immunogenic responses needs the intervention of external stimuli to help the body restore homeostasis and prevent lethality.

## Data Availability Statement

The original contributions presented in the study are included in the article/supplementary material. Further inquiries can be directed to the corresponding author.

## Author Contributions

NK has done primary research and wrote major portions of the manuscript. SK and CK both wrote portions of the manuscript and proofread the manuscript. MJ conceptualized the idea, guided the research, and proofread the manuscript. All authors contributed to the article and approved the submitted version.

## Funding

The research work of this review article is supported by the national institute of health (NIH R01GM133961) grant.

## Conflict of Interest

The authors declare that the research was conducted in the absence of any commercial or financial relationships that could be construed as a potential conflict of interest.

## Publisher’s Note

All claims expressed in this article are solely those of the authors and do not necessarily represent those of their affiliated organizations, or those of the publisher, the editors and the reviewers. Any product that may be evaluated in this article, or claim that may be made by its manufacturer, is not guaranteed or endorsed by the publisher.
